# Contributions of default mode network stability and deactivation to adolescent task engagement

**DOI:** 10.1038/s41598-018-36269-4

**Published:** 2018-12-21

**Authors:** Ethan M. McCormick, Eva H. Telzer

**Affiliations:** Department of Psychology and Neuroscience, University of North Carolina, Chapel Hill, North Carolina 27599 USA

## Abstract

Out of the several intrinsic brain networks discovered through resting-state functional analyses in the past decade, the default mode network (DMN) has been the subject of intense interest and study. In particular, the DMN shows marked suppression during task engagement, and has led to hypothesized roles in internally-directed cognition that need to be down-regulated in order to perform goal-directed behaviors. Previous work has largely focused on univariate deactivation as the mechanism of DMN suppression. However, given the transient nature of DMN down-regulation during task, an important question arises: Does the DMN need to be *strongly*, or more *stably* suppressed to promote successful task learning? In order to explore this question, 65 adolescents (M_age_ = 13.32; 21 females) completed a risky decision-making task during an fMRI scan. We tested our primary question by examining individual differences in absolute level of deactivation against the stability of activation across time in predicting levels of feedback learning on the task. To measure stability, we utilized a model-based functional connectivity approach that estimates the stability of activation across time *within* a region. In line with our hypothesis, the stability of activation in default mode regions predicted task engagement over and above the absolute level of DMN deactivation, revealing a new mechanism by which the brain can suppress the influence of brain networks on behavior. These results also highlight the importance of adopting model-based network approaches to understand the functional dynamics of the brain.

## Introduction

With the advent of resting-state fMRI, there has been an explosion of interest in characterizing intrinsic neural networks, as well as describing their various contributions to human cognition and behavior. Of particular interest to researchers has been the default mode network (DMN), a functionally-related system of regions which show greater metabolic activity at rest compared to task^1^. Regions of the DMN include posterior cingulate, medial-prefrontal, hippocampal, and lateral temporal areas, and are often defined as regions showing strong functional connectivity with the posterior cingulate during rest^2^. While the exact function of the DMN has proven elusive, it is involved in a wide variety of cognitions, such as autobiographical memory, spontaneous thought, and the integration of social information^3–6^. Furthermore, disruption of DMN connectivity is linked to psychopathological states such as schizophrenia, ADHD, conduct disorder, and depression^7–10^.

Importantly, activation in DMN regions shows an inverse relationship with “task-active” regions such as the fronto-parietal and salience networks. These networks show increased activation during task conditions^11,12^, in contrast to the DMN, which often shows strong deactivation (relative to baseline) during decision making (i.e., “task-negative”)^2^. Furthermore, functional anti-correlations (i.e., connectivity) between task-active regions and the DMN are regularly seen during task^11,13^ and are thought to reflect the degree of task engagement and attention^14,15^. However, suppression (i.e., the down-regulation of a network’s influence on behavior, often indexed by deactivation of the BOLD signal) of the DMN by task-related activity appears to be transient^16^, with the default mode network coming back online quickly once task demands ease^15^. Additionally, individuals who exhibit psychopathologies such as ADHD often fail to show DMN suppression^17^, reflecting difficulties in maintaining sustained attention to a task.

One key form of task engagement is feedback learning, during which individuals must maintain mental representations of the task structure and reinforcement history in order to guide future behavior^18–20^. Processes which impact feedback learning are important during adolescence, as teens are particularly sensitive to performance-relevant cues in their environment, showing both developmental^19,21^ and inter-individual^20,22,23^ differences in sensitivity to positive and negative feedback information. Given the role of DMN in disrupting attentional and engagement processes^14,15^, failure to suppress DMN regions during task should be related to decreased feedback learning, as adolescents disengage (as a result of decreased attention and/or motivation) from task information. This disengagement could have particularly important consequences for adolescents, as deficits in the ability to learn from feedback can have negative impacts on adolescent behavior^19,20^.

Given the transient nature of DMN suppression during task, one key unanswered question remains when considering potential mechanisms involved in down-regulating (or suppressing) the default mode network during task engagement: Does the DMN need to show *reduced* (i.e., a decrease in the absolute level of activation), or more *stable* (i.e., reduced fluctuations in activation) activity to achieve suppression? This question distinguishes between the mean level of neural activation (or deactivation as the case may be) and the stability over time of DMN activity as the mechanism by which the brain reduces the influence of functional networks on behavior. Under the first hypothesis, we would expect that individuals who show greater task engagement (indexed by increased feedback learning) would show the greatest DMN deactivation during task. Alternatively, feedback learning may be reflected in more stable DMN activity over time, as suppression of the DMN causes the network to become less responsive to changing task dynamics. Importantly, these two explanations may not be mutually exclusive, as stable and strong deactivation might co-occur.

In the current study, we tested the hypothesis that stability in task-negative regions of the DMN contributes to feedback learning (i.e., participants’ ability to extract and respond to information cues from the task environment), over and above absolute level of deactivation, against the alternative hypothesis that suppression of the DMN is primarily achieved through reductions in univariate activation (i.e., deactivation). To do so, adolescents completed a risky decision-making task, the Balloon Analog Risk Task (BART) during functional magnetic resonance imaging (fMRI). Using an ROI-based approach for both traditional univariate and model-based network analyses, we extracted parameter estimates of DMN deactivation and stability for each individual. We then entered these two types of parameters as simultaneous predictors of adolescents’ engagement on the task. While previous work has focused on the absolute deactivation of DMN, we hypothesized that DMN stability would be a more powerful predictor of feedback learning than absolute level of deactivation in these regions. However, we further predicted that stability and level of deactivation would be positively related, with stronger deactivation being associated with greater DMN stability, suggesting a possible reconciliation of this hypothesis with previous conceptualizations of the DMN during task.

## Methods

### Participants

Sixty-seven adolescent participants completed an fMRI scan. One participant was scanned using the wrong head coil, and another was excluded for excessive movement (>10% of slices with movement in excess of 2 mm), resulting in a final sample of 65 adolescents (*M*_*age*_ = 13.32, *SD* = 0.62, *range* = 12.42–14.83; 56 Caucasian, 2 African American, 7 mixed race/multiple responses). Participants were largely from high income households (1 $0–14,999; 3 $15–29,999; 6 $30–44,999; 4 $45–59,999; 8 $60–74,999; 9 $75–89,999; 30 > $90,000; and 4 not reported), with highly educated parents (3 completed a high school diploma; 8 completed some college; 4 completed an associate degree; 21 completed a bachelor’s degree; 5 completed some graduate school; 18 completed a master’s degree; and 3 completed a professional degree). Written informed assent was obtained for all participants under the age of 18, and written informed consent was obtained from each participants’ parent and/or legal guardian. All methods were carried out in accordance with the relevant guidelines and regulations outlined by the Declaration of Helsinki and experimental protocols were approved by the University of Illinois, Urbana-Champaign Institutional Review Board.

### Risky Decision-Making Task

Participants completed a version of the Balloon Analogue Risk Task (BART), a well-validated experimental paradigm^24,25^ that has been adapted for fMRI in developmental populations^19,26^. The BART measures participants’ willingness to engage in risky behavior in order to earn rewards, and is associated with real-life risk taking in adolescents^20,27^ and adults^24,25^. During the scan session, participants were presented with a sequence of 24 balloons that they could pump up to earn points. Each pump decision was associated with earning one point but also increased the risk that a balloon would explode. If participants pumped a balloon too many times, the balloon would explode and participants would lose all the points they had earned for that balloon. However, if participants chose to cash out before the balloon exploded, the points they earned would be added to the running total of points, which was presented on the screen as a points meter. Participants were instructed that their goal was to earn as many points as possible during the task. Each event (e.g., larger balloon following a pump, new balloon following cashed or explosion outcomes) was separated with a random jitter (500–4000 ms). Balloons exploded after 4 to 10 pumps, and the order of balloons was presented in a fixed order (after being pseudo-randomly ordered prior to data collection), although none of this information was made available to participants. The BART was self-paced and would not advance unless the participant made the choice to either pump or cash out. Participants were told that they could win a $10 gift card at the end of the neuroimaging session if they earned enough points during the task. The point threshold for winning this prize was intentionally left ambiguous so that participants were motivated to continue earning points throughout the task. In reality, all participants were given a $10 gift card after completing the scan session.

### Task Engagement

To measure adolescents’ task engagement during the risky decision-making task, we calculated two indices of feedback learning. Specifically, we were interested in how adolescents used feedback information from previous trials in order to guide their current behavior, and to adapt that behavior when it results in maladaptive outcomes^26^. Previous research using the BART has shown that adolescence is a time of increased feedback learning (compared with childhood), and that individual differences in feedback learning predict differences in risk behavior^19^. In the current study, we were interested in two types of feedback learning to measure task engagement.

First, we estimated how sensitive adolescents were to the valence of feedback on the task. To do so, we measured the impact of experiencing positive (i.e., a cash-out) versus negative (i.e., an explosion) on the previous trial. For this metric, larger positive values indicate that adolescents increase their pumping behavior following positive feedback and decrease pumping following negative feedback, while values close to zero indicate pump behavior that is random with respect to the valence of feedback that adolescents receive. Secondly, we estimated adolescents’ sensitivity to the value (i.e., magnitude) of feedback on the previous trial, by contrasting risk decisions made after receiving low-value feedback (i.e., earning or losing points on a small or medium-sized balloon) versus those made after high-value feedback (i.e., earning or losing points on relatively large balloons). Larger positive values on this metric indicate that adolescents change their pump behavior more after a high-value feedback event, whereas values close to zero indicate that adolescents’ decisions to pump were not impacted by the value of points earned (in a previous cash-out) or lost (in a previous explosion). Each of these feedback learning indices measure how adolescents retain relevant task information to guide their ongoing risk behavior.

To obtain these indices, we took a multi-level modeling approach utilizing the SAS software package (SAS version 9.4; SAS Institute Inc., Durham, NC), in which trials (24 balloons) were nested within adolescents, and the level 1 outcome was the final number of pump decisions made on a given balloon. To obtain our learning indices, we modeled pump number at the trial level as dependent on (1) previous feedback and (2) the size of that feedback. Consistent with previous research^27,28^, we also controlled for the overall trial number and the outcome of the current balloon, resulting in the following Level 1 equation:

$$\begin{array}{c}Number\,of\,Pump{s}_{ij}={\gamma }_{0j}+{\gamma }_{1j}Trial\,Numbe{r}_{ij}+{\gamma }_{2j}Current\,Outcom{e}_{ij}\\ \,+\,{\gamma }_{3j}Previous\,Outcome\,Valenc{e}_{ij}+{\gamma }_{4j}Previous\,Outcome\,Valu{e}_{ij}+{\mu }_{0j}+{\varepsilon }_{ij}\end{array}$$

Total pumps on a particular balloon trial (*i*) for a given adolescent (*j*) was modeled as a function of the average number of pumps across the task (*γ*_0*j*_), the trial number (*γ*_1*j*_; *range* = 0–23), the outcome of the current trial (*γ*_2*j*_; coded Cash-Out = 0, Explosion = 1), the outcome of the previous trial (*γ*_3*j*_; coded Cash-Out = 0, Explosion = 0), and the size of the previous outcome (*γ*_4*j*_), in which we calculated a 75th percentile threshold for each participant’s pump behavior based on their individual data. For previous trial outcomes on balloons where adolescents pumped above this threshold, the predictor was coded as 1, as points earned or lost on these trials were high value for the individual, while all other previous trials were coded as 0, indicating lower value earnings or loss^28^. As our focus was on adolescents’ individual sensitivity to the valence and value of previous feedback, these parameters were allowed to vary randomly in our model in order to gain individual effect estimates. Nesting of trials within balloons was achieved by modeling a between-person random intercept (*μ*_0*j*_) assumed to be independent and identically distributed and follow a Normal distribution with a constant variance (i.e., ${u}_{0j} \sim {\rm{N}}[0,{\tau }_{00}]$). Finally, the individual-level residuals errors (ε_*ij*_) were assumed to be independent and identically distributed, following a Normal distribution with a constant variance (i.e., ${\varepsilon }_{ij} \sim {\rm{N}}[0,{\sigma }^{2}]$). In order to use these two metrics of task engagement, we extracted empirical Bayes estimates for each adolescent. Empirical Bayes estimates are optimally-weighted averages which combine individual- and group-level slope estimates, and “shrink” individual slope estimates towards group mean effect^29^.

### fMRI Data Acquisition and Processing

#### fMRI data acquisition

Imaging data were collected utilizing a 3 Tesla Trio MRI scanner. The BART included T2*-weighted echoplanar images (EPI; slice thickness = 3 mm; 38 slices; TR = 2 sec; TE = 25 ms; matrix = 92 × 92; FOV = 230 mm; voxel size = 2.5 × 2.5 × 3 mm^3^). Additionally, structural scans were acquired, including a T1* magnetization-prepared rapid-acquisition gradient echo (MPRAGE; slice thickness = 0.9 mm; 192 slices; TR = 1.9 sec; TE = 2.32 ms; matrix = 256 × 256; FOV = 230 mm; voxel size = 0.9 × 0.9 × 0.9 mm^3^; sagittal plane) and a T2*-weighted, matched-bandwidth (MBW), high resolution, anatomical scan (slice thickness = 3 mm; 192 slices; TR = 4 sec; TE = 64 ms; matrix = 192 × 192; FOV = 230 mm; voxel size = 1.2 × 1.2 × 3 mm^3^). EPI and MBW scans were obtained at an oblique axial orientation in order to maximize brain coverage and minimize dropout in orbital regions.

#### fMRI data preprocessing and analysis

Preprocessing utilized FSL FMRIBs Software Library (FSL v6.0; https://fsl.fmrib.ox.ac.uk/fsl/). Steps taken during preprocessing included correction for slice-timing using MCFLIRT; spatial smoothing using a 6 mm Gaussian kernel, full-width-at-half maximum; high-pass temporal filtering with a 128 s cutoff to remove low frequency drift across the time-series; and skull stripping of all images with BET. Functional images were re-sampled to a 2 × 2 × 2 mm space and co-registered in a two-step sequence to the MBW and the MPRAGE images using FLIRT in order to warp them into the standard stereotactic space defined by the Montreal Neurological Institute (MNI) and the International Consortium for Brain Mapping. Preprocessing was completed utilizing individual-level independent component analysis (ICA) with MELODIC combined with an automated component classifier^30^ (Neyman-Pearson threshold = 0.3), which was applied to filter signal originating from noise sources (e.g., motion, physiological rhythms). Global signal regression was not performed due to its tendency to increase distance-related dependencies in the strength of functional connectivity measures^31^.

#### Motion Correction

Prior to modeling the fMRI data further, we took several steps to reduce the influence of motion. First, as mentioned previously, we subjected each participants’ data to individual-level ICA in order to remove motion-related signal from each participants’ time-series. We also controlled for 8 nuisance regressors in the GLM and time-series analyses: 6 motion parameters generated during realignment and the average signal from both the white matter and cerebrospinal fluid masks. Finally, slices with greater than 2 mm of motion were censored from the time-series (or modeled as a junk regressor in the GLM) to remove the effects of large, sudden movements on the functional data. No participant exceeded 5% of slices being censored (range: 0–2.5%). Previous work has shown that these strategies effectively reduce the influence of motion on functional connectivity analyses^31^.

### Regions of Interest

**Figure 1:**
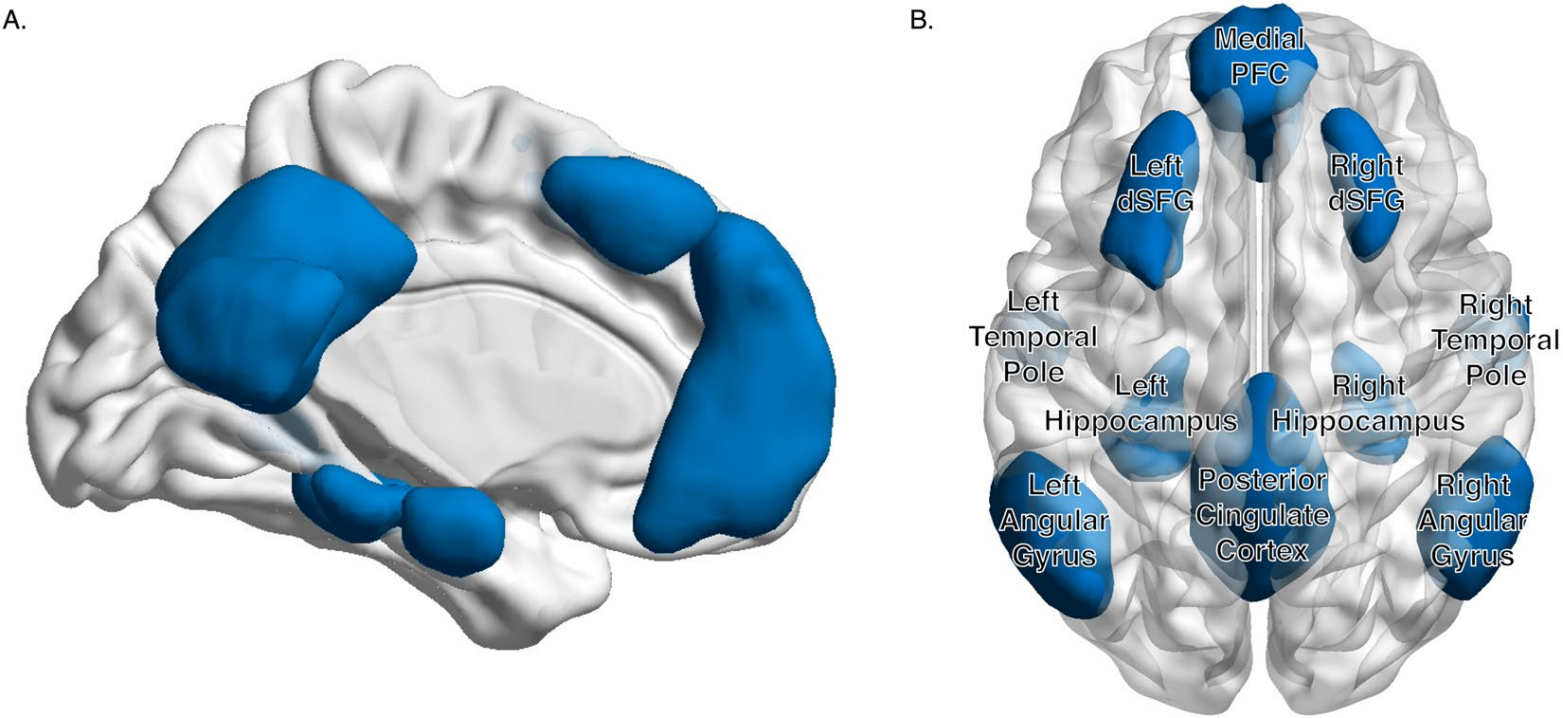
Default Mode Network. We defined 10 ROIs composing regions central to the default mode network (DMN). (**A**) A 3D video of our *a priori* regions of interest in free space. (**B**) Regions included the medial prefrontal cortex, posterior cingulate cortex, and bilateral complements of the dorsal superior frontal gyrus (dSFG), temporal pole, hippocampus, and angular gyrus.

To estimate how autoregressive stability in the default mode network impacts task behavior, we constructed 10 *a priori* regions of interest (ROIs) based on previous neuroimaging work with this network (Fig. 1). We based our ROIs off of resting-state maps of the default mode network^32,33^. Regions included the medial prefrontal cortex (mPFC), posterior cingulate cortex (PCC), bilateral dorsal superior frontal gyrus (dSFG), bilateral temporal poles (TP), bilateral hippocampus, and bilateral angular gyrus (AG). Regions were extracted from the templates using FSL. Most ROIs showed good separation from other DMN regions, however, in order to separate the mPFC and bilateral dSFG (which overlap in standard maps), we took additional steps to create 3 separate ROIs by zeroing voxels between the two regions with a z-stat < 6. This approach achieved appreciable separation for our masks. Individual masks were then evaluated again using the Marsbar toolbox in SPM^34^ and FSL to ensure that ROIs did not contain any voxels that overlapped with another mask or exceeded the boundaries of the whole-brain mask. A 3D, navigable image containing all masks superimposed onto a single brain map is available on NeuroVault (https://neurovault.org/collections/VSWQSTDA/ ^35^.

### Whole-brain Univariate Analyses

For our univariate analyses, we modeled the BART as an event-related design. Whole-brain statistical analyses were performed using the general linear model (GLM) in SPM8. Fixed effects models were constructed for each participant with several conditions of interest, including pump decisions, cash-outs, and explosion events. The jittered inter-trial period between pump decisions and between outcomes and a new balloon was not modeled and served as an implicit baseline. A parametric modulator (PM) was included for conditions of interest and corresponded to the current pump number for the current trial at the time of event. This PM serves to control for differences across pumps within a balloon. For descriptive purposes, we ran whole-brain, group-level, random effects analyses for pump decisions using GLMFlex (http://mrtools.mgh.harvard.edu/index.php/GLM_Flex). This approach corrects for variance-covariance inequality, removes outliers and sudden activation changes in the brain, partitions within- and between-person error terms, and analyzes all voxels containing data. Since this analysis was purely for visualization purposes, we thresholded the resultant statistical image at *p* < 0.001, with a minimum cluster size of 40 voxels. For the central univariate analyses, we extracted parameter estimates from each individual’s unthresholded fixed effects statistical map using the 10 *a priori* ROIs we constructed for the DMN network.

### Time-series Analysis

#### Granger Causality

Originally developed in the context of economic models^36^, Granger causality emerges from a vector autoregression framework (VAR), where the contemporaneous and lagged relationships between a system of variables can be examined. A weak form of causal inference (compared to experimental designs for example), Granger causality relies on the intuition that *x* cannot cause *y* if *x* temporally follows *y* (i.e., cause precedes effect)^37^. Based on this idea, Granger causality can be inferred if *x* at a previous time point (e.g., *t* − 1) predicts *y* at time (*t*) above and beyond the self-predictive effect of *y* at *t* − 1 on *y* at *t*. In other words, the combined information of *x*_(*t*−1)_ and *y*_(*t*−1)_ is more predictive of *y*_(*t*)_ than is *y*_(*t*−1)_ alone. Under this causal definition, it is possible for variables to Granger cause one another across time^38^.

#### Group Iterative Multiple Model Estimation (GIMME)

GIMME is a model-based network approach, which utilizes both individual and group-level information to derive directed functional connectivity maps^39^. GIMME estimates connectivity graphs using both unified SEM^40^ and extended unified SEM^41^ to assess whether the presence of a path between ROIs significantly improves the overall model fit to the time-series data. GIMME estimates both contemporaneous (e.g., ROI_1_ at *t* predicts ROI_2_ at *t*) and lagged (e.g., ROI_1_ at *t* − 1 predicts ROI_2_ at *t*) effects between ROIs, as well as the autoregressive (e.g., ROI_1_ at *t* − 1 predicts ROI_1_ at *t*) effects for each ROI time-series. Formally, a GIMME model for a set of *p* time-series with *t* measurements is:

$${\eta }_{i}(t)=({A}_{i}+{A}_{{i}^{g}}){\eta }_{i}(t)+({{\rm{\Phi }}}_{1,i}+{{\rm{\Phi }}}_{1,{i}^{g}}){\eta }_{i}(t-1)+{\zeta }_{i}(t)$$

where *A* represents a *p* × *p* matrix of contemporaneous paths for the individual (*A*_*i*_) and group (${A}_{{i}^{g}}$) parameters, Φ_1_ is matrix of first-order lagged paths (for the individual and group respectively), and *ζ* is the *p*-length vector of errors, assumed to be a white noise process, with means of zero, a finite variance, and no sequential dependencies (i.e., all temporal information is contained within *A* and Φ_1_)^42^. The diagonal of Φ_1_ contains path estimates for the autoregressive effects (e.g., ROI_1_ at *t* − 1 predicts ROI_1_ at *t*), which represent the autocorrelations of each ROI predicting itself forward in time. GIMME assesses directional paths by testing whether a given ROI can predict another, controlling for the predicted ROI’s autoregressive effect (i.e., establishing Granger causality). GIMME has been developed for both block^40^ and event-related^41^ fMRI data, and is freely available through the open-source R platform^43^.

In contrast with many other functional connectivity approaches (e.g., graph theoretical approaches), GIMME constructs functional maps through a model-driven, multi-step processing of model building and pruning. First, information across all participants is used to derive a common network map that is representative of the majority of the sample. Group paths are only retained if they are significant for 70% of all individuals in the sample. All autoregressive paths are automatically estimated in order to accurately assess directionality in the between-ROI paths. Once a group map has been obtained, additional paths at the individual level are evaluated based on improvements to model fit for that individual. Unnecessary paths are pruned at the group level, and additional paths at the individual level are evaluated based on improvements to model fit for that individual. Individual-level paths are then pruned if they do not significantly improve the fit of the final model. This approach offers the unique advantage of being able to derive a group-level map that should be applicable to the majority of the sample, while still recognizing that individuals often show significant heterogeneity from the group map. This approach shows significant advantages over other methods in recovering “true” paths in simulated data while minimizing false positives^44^.

Our task provided two main challenges when measuring neural connectivity. First, our goal was to analyze connectivity patterns during risky decisions; however, the BART also contains feedback trials (i.e., cash-out outcomes, explosions). Secondly, our task was self-paced and as such, we needed a modeling approach that would allow for individuals to possess different amounts of data. Fortunately, GIMME is capable of handling unequal amounts of data between participants, as well as the inclusion of missing data^45^. Missing values are replaced with placeholder NaN values to maintain the temporal ordering of scans, and neither contemporaneous nor lagged effects are estimated based on missing values. These features make GIMME especially well-suited to estimating connectivity graphs for the BART, allowing for the self-paced nature of the task, as well as specifically examining connectivity during risk decisions, without considering connectivity during outcomes.

#### Autoregressive Paths

Autoregressive pathways are estimated as the predictive effect of activity in an ROI at one time point on that ROI’s activity at the next time point. As such, stronger autoregressive path indicate that an ROI’s activation is more stable over time. In our analyses, we were specifically interested in testing whether the strength of an individual’s autoregressive paths was related to task behavior on the BART. As such, the parameter estimates from GIMME for each ROI’s autoregressive path were extracted for use in subsequent regression analysis.

### Analytic Plan

To test the hypothesis that stability versus deactivation of the default mode network would be important for feedback learning, we took two analytic approaches. First, we utilized standard univariate analyses to extract each individual’s parameter estimates of deactivation in the 10 *a priori* DMN ROIs during risk decisions. Secondly, we took a model-based network approach using the same 10 ROIs to estimate stability over time in DMN activation. After completing both univariate and network analyses, each participant had 10 parameter estimates of mean activation and 10 parameter estimates of stability in activation over time. To avoid concerns of multiple comparisons by regressing each of these 20 estimates on behavioral metrics of interest, we took a dimension reduction approach through principal components analysis (PCA). PCA also offers a key advantage by partitioning variance into bins: variance that is common across regions (and representative of the DMN as a network), and variance that is unique to a particular region. Because activation in a given region is likely a combination of network- and ROI-level information, partitioning this region-specific variance out helps remove noise from our estimates that originate from individual ROIs. For both sets of parameters, we extracted the first principal component from a PCA where estimates from all 10 ROIs were used as inputs. We utilized the R function, “principal” (https://cran.r-project.org/web/packages/psych/psych.pdf), to extract the first principal component utilizing the covariance matrix and varimax rotation. We ran follow-up analyses using the cross-validation function in the R function “pca” (https://cran.r-project.org/web/packages/mdatools/index.html), and results remained unchanged. These principal component scores were then used in subsequent regression analyses to predict task engagement (i.e., sensitivity to the valence and value of feedback in the task).

## Results

### Group-Level Results

#### DMN Deactivation During Risk Decisions

**Figure 2:**
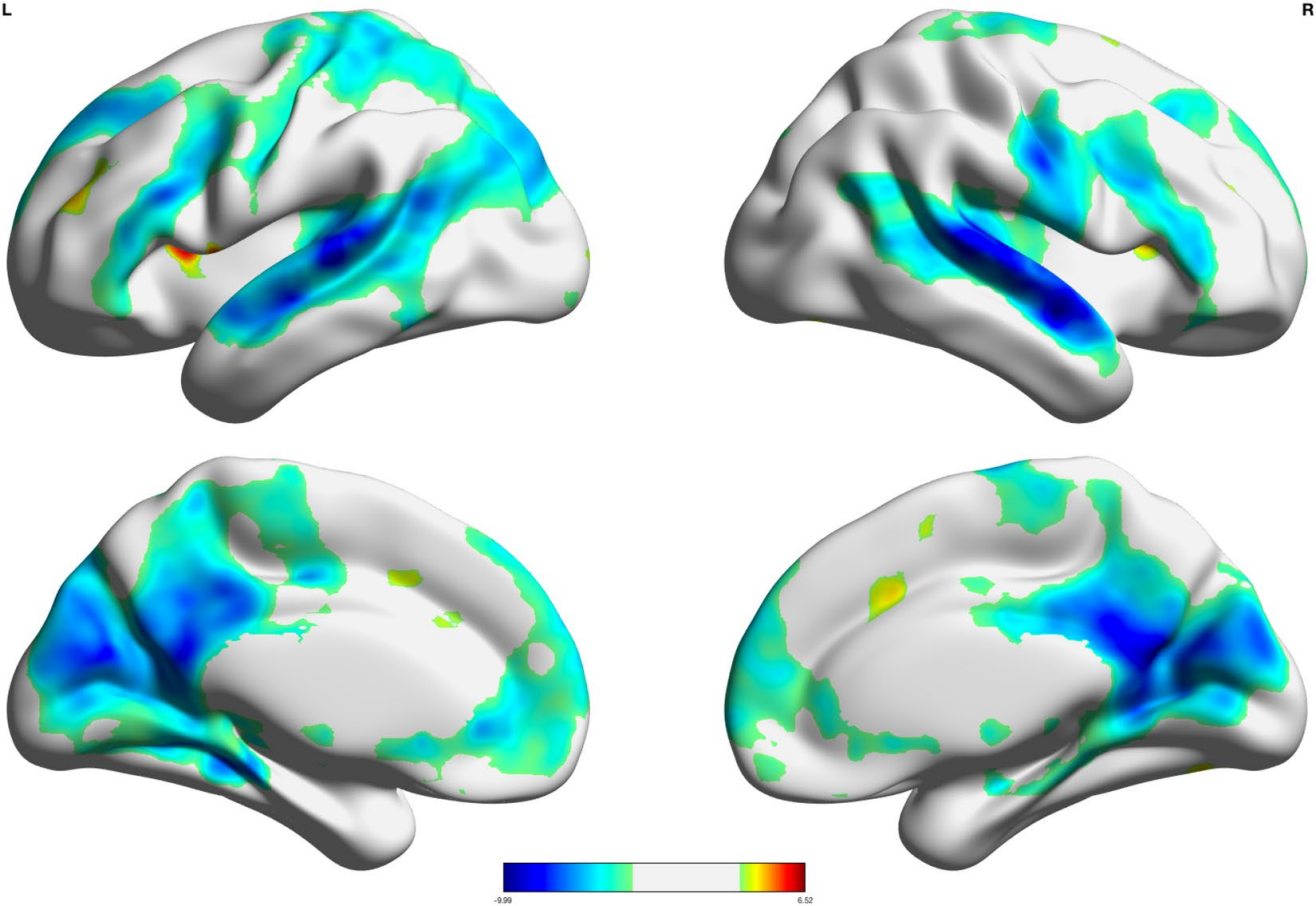
Main Effect of Risk Decisions. The univariate condition of risk decisions showed robust deactivation across all DMN regions. Salience regions, such as the anterior cingulate and anterior insula showed strong positive activation.

**Figure 3:**
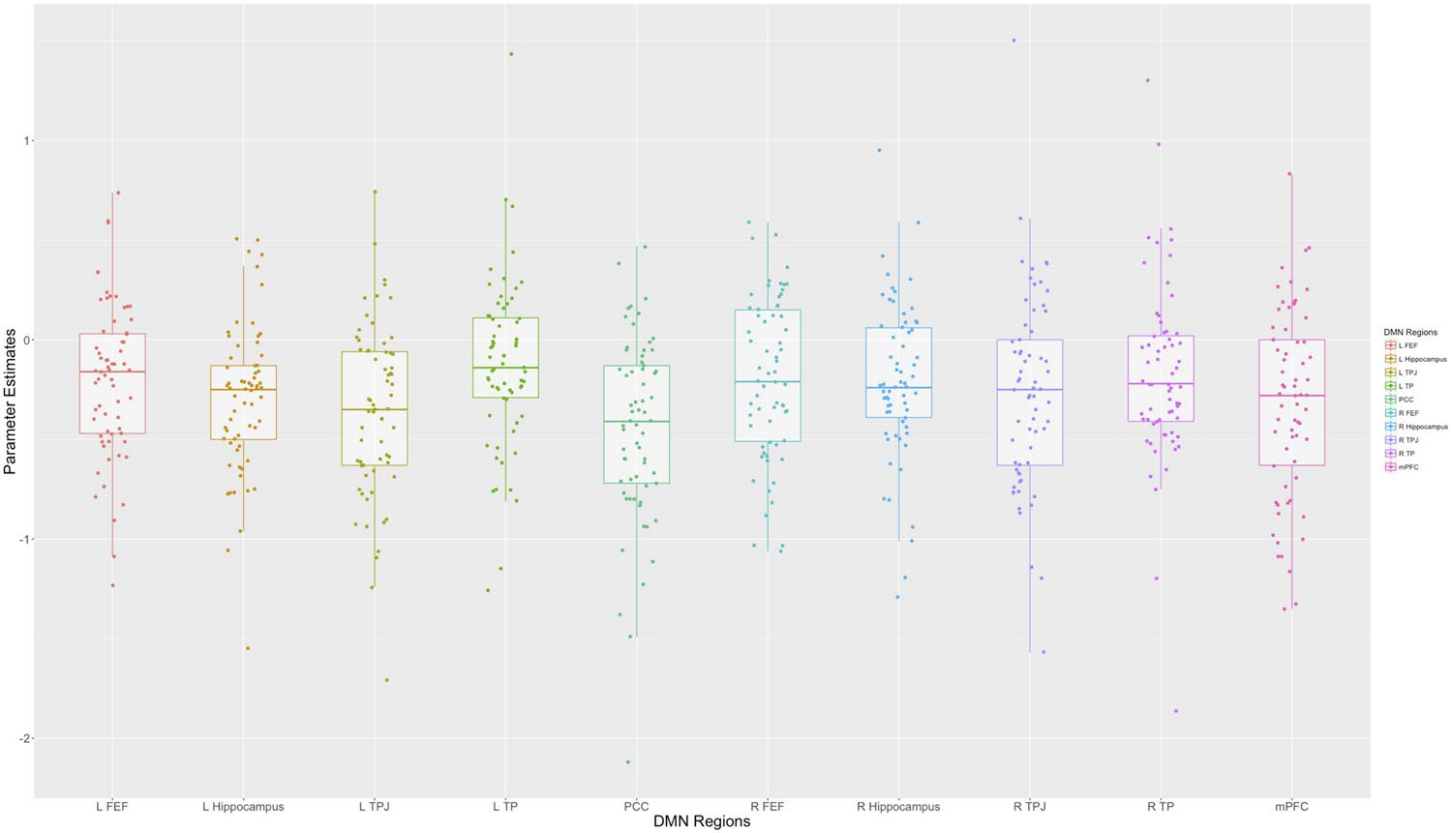
Distribution of Activation and Deactivation in the DMN. While regions of the default mode network show mean deactivation at the group-level, there are individual differences, including individuals who show positive activation of the DMN on average.

We first ran main effects analysis at the whole brain level, for descriptive purposes, to check for the expected deactivation of the default mode regions during decisions to pump across individuals in the sample. Consistent with prior work^2^, adolescents showed strong deactivation of default mode regions during risk decisions at the group level, including mPFC, PCC, STS, AG, dSFG, and hippocampus (Fig. 2; Table 1). In contrast, typical task-active regions such as the anterior cingulate, anterior insula, and motor cortex showed positive activation during risk decisions. However, substantial individual differences emerged such that not all adolescents showed strong deactivation of DMN regions, and some adolescents even showed positive activation of DMN regions during risk decisions (Fig. 3).

**Table 1:** Neural Regions Showing Significant Activation During Risky Decisions (i.e., Pumps).

Anatomical Region	+/−	BA	x	y	z	t	k
*“Task-Positive” Regions*							
ACC	+	24/32	−4	24	30	4.69	514
L Anterior Insula	+		−34	14	10	6.52	530
R Anterior Insula	+		34	20	8	4.39	42
L MFG	+	9/46	−32	42	18	4.53	364
R MFG	+	9/46	28	42	22	3.91	48
R Putamen	+		22	8	−8	4.32	109
L Cerebellum	+		−36	−50	−32	5.99	341
R Cerebellum	+		38	−56	−28	5.97	773
“*Task-Negative*” *Regions*							
R MTG^a^	−	21	56	−4	−12	9.99	18365
R TP^a^	−	38	58	4	−12	9.52	
R STG^a^	−	22	62	−28	12	8.67	
L STG^a^	−	22	−58	−28	6	9.38	
L dSFG^a^	−	8	−18	36	46	6.12	
R dSFG^a^	−	8	20	22	42	6.85	
L IFG (triangularis)^a^	−	45	−48	20	24	7.20	
R IFG (triangularis)^a^	−	45	40	16	24	6.26	
L IFG (orbitalis)^a^	−	47	−54	32	10	5.79	
R IFG (orbitalis)^a^	−	47	54	36	6	7.35	
L Lateral OFC^a^	−	11	−40	34	−10	4.26	
R Lateral OFC^a^	−	11	30	34	−12	5.75	
L Hippocampus^a^	−		−28	−40	−14	7.90	
R Hippocampus^a^	−		22	−38	−12	4.58	
Rectal Gyrus^a^	−	11	0	18	−6	5.60	
Medial OFC^a^	−	11	−4	42	−6	5.35	
vmPFC^a^	−	10/11	2	58	−2	5.15	
amPFC^a^	−	10	−1	65	8	4.62	
dmPFC^a^	−	9	8	60	28	5.09	
Precuneus^b^	−	7	8	−56	18	9.23	32480
L TP^b^	−	38	−52	−16	−8	8.57	
Calcarine Gyrus^b^	−	17	−6	−70	20	8.45	
PCC^b^	−	29/30	6	−46	26	8.12	

*Note:* L and R refer to left and right hemispheres; + and − refer to positive or negative activation; BA refers to Brodmann Area of peak voxel; k refers to the number of voxels in each significant cluster; t refers to peak activation level in each cluster; x, y, and z refer to MNI coordinates. Superscripts (e.g. a, b, etc.) indicate that peak voxels are part of a contiguous cluster. mPFC = Medial Prefrontal Cortex; ACC = Anterior Cingulate Cortex, MFG = Middle Frontal Gyrus, MTG = Middle Temporal Gyrus, TP = Temporal Pole, STG = Superior Temporal Sulcus, dSFG = Dorsal Superior Frontal Gyrus, IFG = Inferior Frontal Gyrus, OFC = Orbitofrontal Cortex, vmPFC = Ventromedial Prefrontal Cortex, amPFC = Anterior Medial Prefrontal Cortex, dmPFC = Dorsomedial Prefrontal Cortex, PCC = Posterior Cingulate Cortex.

#### Network Map of the DMN During Risk Decisions

**Figure 4:**
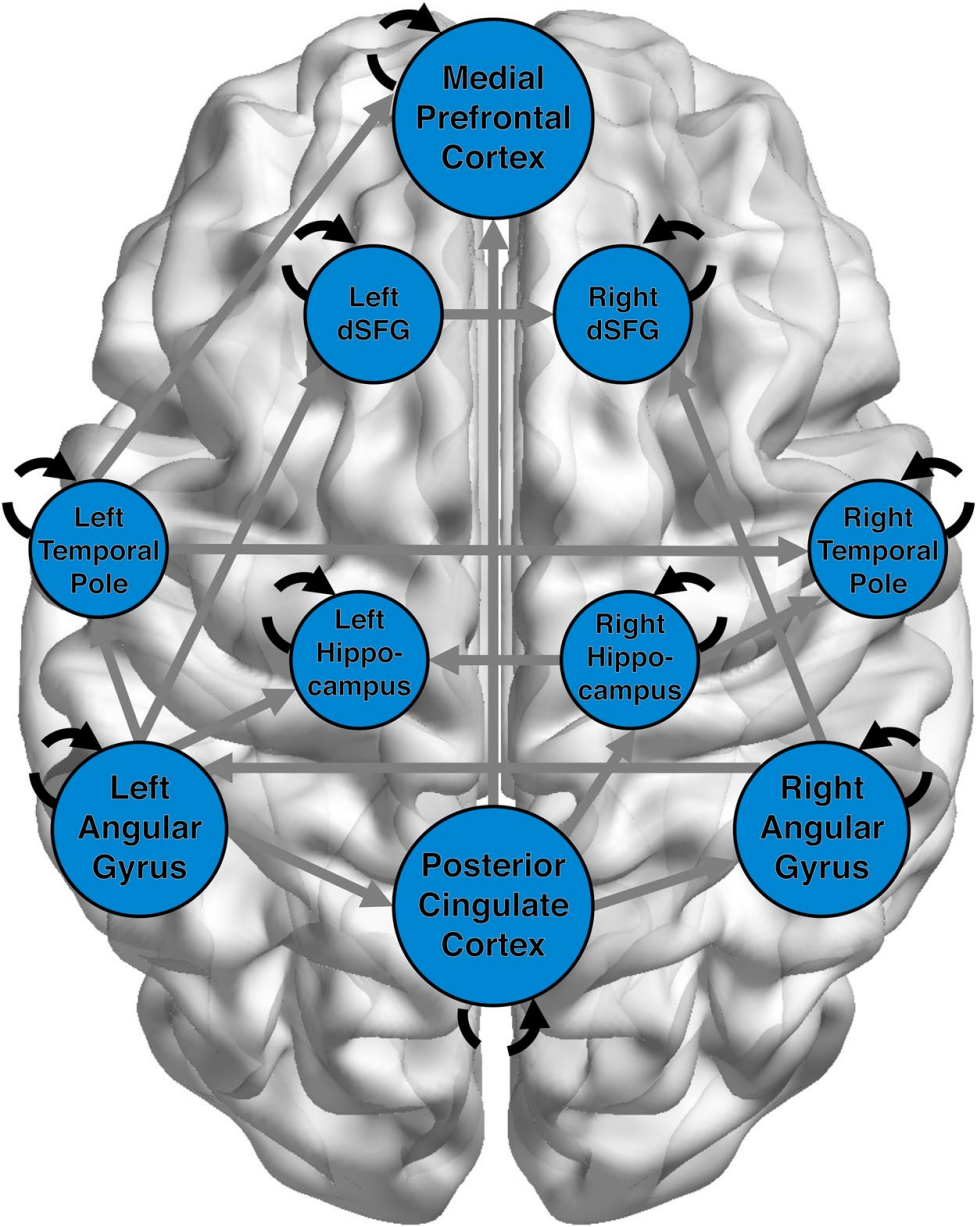
DMN Network during Risk Decisions. DMN seed regions showed strong interconnectivity (grey), with hubs such as the left angular gyrus and posterior cingulate showing several cross-region connections. However, for the purpose of the current study, our main focus was the autoregressive paths (black) which are estimates of within-region stability in activation. Autoregressive paths are dashed to denote a lagged temporal relationship.

Next, we constructed model-based functional networks between our *a priori* DMN ROIs. While our focus was on the autoregessive paths, we estimated and displayed the full group model for descriptive purposes. Results show a strongly interconnected default mode network (Fig. 4). In addition to connections between bilateral complements (e.g., left and right dSFG), the PCC, left TP, and left AG show many between-region paths. Importantly for testing our hypothesis, the autoregressive pathways for each of the 10 DMN ROIs were estimated for all subjects.

#### Individual Differences in DMN Deactivation and Stability Differentially Predict Feedback Learning

Finally, our key analysis explored individual differences in deactivation (from the univariate analyses) and stability (from the functional network analyses) in DMN regions to task engagement. We took parameter estimates of univariate activation and autoregressive strength for each of the 10 *a priori* DMN regions and ran separate PCA analyses on each type of parameter and obtained one score per person, per analysis. For univariate analyses, this analysis resulted in a representative level of deactivation across DMN regions. For network analyses on the autoregressive paths, the PCA score reflected representative activational stability in the DMN as a whole (see Table 2 for factor loadings to each principal component).

**Table 2:** Factor Loadings for Regions of DMN on Principal Components for Mean Levels of Deactivation and Autoregressive Stability.

Factor Type	DMN Region	Rescaled Factor Loading
*Deactivation*		
	Left dSFG	0.758
	Left Hippocampus	0.741
	Left AG	0.877
	Left TP	0.662
	PCC	0.892
	Right dSFG	0.623
	Right Hippocampus	0.691
	Right AG	0.770
	Right TP	0.760
	mPFC	0.828
*Autoregressive Strength (Stability)*		
	Left dSFG	0.744
	Left Hippocampus	0.025
	Left AG	0.341
	Left TP	0.514
	PCC	0.768
	Right dSFG	0.644
	Right Hippocampus	0.205
	Right AG	0.594
	Right TP	0.229
	mPFC	0.633

Next, we entered both of the scores into a multiple regression analysis with adolescents’ two indices of feedback learning (i.e., sensitivity to the valence and sensitivity to the value of previous feedback) as our outcomes in two separate analyses (thresholded at *p* = 0.025 to correct for multiple comparisons). Results showed that DMN stability (*B* = 0.063, *SE* = 0.026, *p* = 0.018) but not mean deactivation (*B* = 0.025, *SE* = 0.026, *p* = 0.349), is associated with adolescents’ sensitivity to the valence of the previous outcome. Similarly, stability (*B* = 0.150, *SE* = 0.048, *p* = 0.003) but not deactivation (*B* = 0.033, *SE* = 0.048, *p* = 0.487) is associated with adolescents’ sensitivity to the value (i.e., magnitude) of previous feedback on the task. Furthermore, the two factor scores were uncorrelated (*r* = 0.112, *p* = 0.376), meaning that deactivation did not indicate more stability in DMN activity, nor were interactions between deactivation and stability predictive of feedback learning (*p* = 0.186 and *p* = 0.963 respectively). These results suggest that stability in DMN activity, even if that activity is positive on average (as is characteristic of some adolescents; Fig. 3), is more predictive of adolescents’ feedback learning than absolute level of (de)activation.

## Discussion

The exact role of the default mode network in cognition and behavior remains an important open question for cognitive neuroscientists. Traditionally, the DMN has been conceptualized as a “task negative” network^1^, with suppression of the network being important for normal decision-making processes and task engagement^2,14^. Indeed, DMN suppression is an important marker of task engagement^14^, showing linear deactivation as the difficulty of the task increases^15^. Furthermore, disruption of DMN suppression is thought to contribute to attention-related disorders such as ADHD^7^. However, unanswered questions remain as to the mechanism by which DMN suppression is implemented in the brain. We utilized both traditional univariate, as well as a novel, model-based network approach to test two competing hypotheses related to this mechanism of DMN suppression. Consistent with previous work^1,2^, main effects analyses showed characteristic deactivation in DMN regions during task. Furthermore, the group connectivity map revealed large numbers of connections between central DMN nodes (e.g., PCC and angular gyrus) and the other nodes of the network. We then used the overall level of DMN deactivation and stability in DMN activation across time to assess whether the absolute level or stability in DMN activation was related to task engagement, operationalized as adolescents’ ability to extract and use feedback information learned from the task environment.

In line with our hypothesis, the stability of activation in default mode regions (as estimated by the autoregressive pathways in GIMME) predicted both metrics of feedback learning over and above the absolute level of DMN deactivation (as measured through the univariate contrast). Indeed, adolescents’ mean level of deactivation in DMN was not a significant predictor of either metric of feedback learning within the task. Interestingly, DMN deactivation was uncorrelated with the stability within those regions, suggesting that highly stable DMN activation was possible even when the DMN showed positive activation during the task. These results suggest that the brain may be able to suppress the influence of neural regions during a task without an apparent change in resource consumption (at least as measured by the BOLD signal).

The implications of the current study offer promise for future research for two reasons. First, the current results offer a validation for the adoption of model-based network approaches for functional data. Traditional approaches to functional connectivity (e.g., seed-based, graph theoretical) only consider concurrent relationships between ROIs. However, methods such as GIMME^41^ and other vector autoregression^40,46^ (VAR) models are capable of estimating both concurrent and lagged effects, which improve network model fit for each individual. Importantly for the current study, GIMME automatically estimates autoregressive paths (i.e., lagged effects within an ROI) as part of its model-building approach, allowing us to examine the temporal stability of activation across time. Our finding that activational stability, as measured through these autoregressive paths, is key for promoting feedback learning highlights the importance of considering these lagged effects and provides encouragement for an increased focus on model-based network approaches that can estimate them.

Secondly, the implication of the current results (i.e., that network influence can be suppressed through stability rather than through deactivation) raises questions about the inferences made about negative BOLD estimates (i.e., deactivation) in fMRI. While deactivation is often viewed as synonymous with a reduced role in decision making processes, the fact that deactivation is not correlated with stability suggests that a highly-deactivated region can still show low stability in activation across the task. Furthermore, we found unexpected variability in the mean level of deactivation in the DMN at the main effect level, such that some adolescents showed the expected pattern of strong DMN deactivation whereas others showed weak deactivation or even positive activation. This suggests that deactivation of the DMN may not be a universal phenomenon during decision making, and that a failure to deactivate DMN does not *a priori* impair performance on the task. Whether there are differences in the behavioral profiles associated with activation fluctuations between individuals who show strongly versus weakly deactivated DMN remains an open question, as the current sample is likely underpowered to detect interaction effects. Future research may be able to address this by examining the interaction between stability and level of deactivation in the DMN, and the consequences of different configurations (e.g., low stability and strong deactivation versus high stability and strong deactivation) for task behavior.

For future research, unanswered questions to consider are the potential mechanisms by which the brain instantiates stability in activation in the DMN regions. One possibility is that task-relevant regions (e.g., ACC, insula) or some third set of regions actively suppresses DMN involvement during task by producing signals which down-regulate default mode regions. Alternatively, other networks could simply disengage from DMN regions. By increasing the segregation between networks, the brain may isolate the DMN, decreasing its ability to influence cognition and behavior. Uncovering the mechanism that instantiates DMN suppression is important for understanding both normal cognition but also has implications for disease states which are associated with disruptions to the DMN^7,8,47^.

In conclusion, we tested two competing hypotheses related to the suppression of the default mode network during a risky decision-making task. In contrast with a focus on the mean level of deactivation, we proposed that stability in activation, rather than absolute level, would be a more-important mechanism for reduced DMN influence on feedback learning. We adopted both traditional univariate and a novel model-based network approach to test these hypotheses, and found support for our hypothesis that increased DMN stability is related to increased sensitivity to information from the task (i.e., learning). These results shed light on a new mechanism by which the brain reduces the influence of a functional network, and highlights the importance of adopting network methods which consider both contemporaneous and lagged effects.

## Electronic supplementary material

Figure 1
